# The Death of Dr. Emile Magitot

**Published:** 1897-07

**Authors:** 


### THE DEATH OF DR. EMILE MAGITOT.

The death-roll of distinguished men in the dental profession has of recent years grown to large proportions, but the name of Dr. Magitot stands pre-eminent, and will pass down into dental history along with that of Sir John Tomes, the two leading scientific minds of the century,—the one in France and the other in England.

The work of Magitot has for so long a period been a part of the intellectual and scientific life of his profession that the dental world has ceased to regard him as belonging exclusively to France, for he had become a recognized authority throughout the world.

His interest in the progress of dentistry remained unabated notwithstanding his advanced age, and we find him in his later years active in the Stomatological Society of Paris, the name of which received his cordial endorsement, if it had not its origin in his active scientific mind.

Dentistry owes to France a large debt for its earliest and best beginnings, but among the brilliant galaxy of names that have adorned its history, in that country, no one will stand higher or be more frequently quoted, as the years go by, than the name of Magitot.

The interesting and appreciative tribute to his life and work, from the pen of Dr. Pietkiewicz, has been translated for the Journal, and will be found under the proper heading. It is worthy the man and the work of his unselfish life.

